# Inferior Vena Cava-Atrial Anastomosis in Liver Transplant Recipient with Inferior Vena Cava Occlusion: A Case Report and Literature Review

**DOI:** 10.3390/jcm15010384

**Published:** 2026-01-05

**Authors:** Jakub Rochoń, Piotr Kalinowski, Joanna Marczak, Krzysztof Gibiński, Michał Grąt

**Affiliations:** 1 Department of General, Transplant and Liver Surgery, Medical University of Warsaw, Banacha 1a st., 02-097 Warsaw, Poland; kuba.rochon@gmail.com (J.R.); joasia.marczak5@gmail.com (J.M.); michal.grat@wum.edu.pl (M.G.); 2 II Department of Clinical Radiology, Medical University of Warsaw, 02-097 Warsaw, Poland; krzysztof.gibinski@uckwum.pl

**Keywords:** inferior vena cava thrombosis, liver transplantation, autoimmune hepatitis, primary biliary cholangitis, Budd-Chiari syndrome, surgical technique

## Abstract

A 25-year-old woman with decompensated liver cirrhosis and complete inferior vena cava (IVC) occlusion was referred to our department for liver transplantation. The etiology of cirrhosis was Budd-Chiari syndrome (BCS) related to systemic lupus erythematosus, autoimmune hepatitis, and primary biliary cholangitis (AIH-PBC) overlap syndrome. Transplantation was feasible due to an extensive collateral circulation of pre-vertebral veins that drained blood from the lower extremities and both kidneys to the azygos-hemiazygos veins. This venous anomaly enabled the excision of the obstructed retrohepatic IVC, followed by an alternative anastomosis of the suprahepatic IVC to the right atrium without reconstruction of the infrahepatic IVC. Despite good venous patency and normalization of liver graft function, the patient developed cecum perforation, cardiovascular and respiratory insufficiency, which led to the patient’s death two months after transplantation. This case report supports an individual approach and highlights the feasibility of liver transplantation despite an extensive IVC thrombosis. To our knowledge, it is the first description of the application of a deceased donor liver transplantation in patients with AIH-PBC overlap syndrome and lupus-related BCS. A concise review of published literature on IVC-atrial anastomosis in adult liver transplant recipients is provided, and the technique is discussed based on our recent experience.

## 1. Introduction

Inferior vena cava (IVC) obstruction is a rare entity in liver transplant candidates, but it may be a limiting factor for transplantation in the most severe cases. Extensive obstruction of the IVC restricts the use of routine surgical techniques and may constitute a contraindication to liver transplantation (LTx). Current guidelines do not address the management of a complete IVC obstruction [1]. LTx with anatomical reconstruction of the hepatic vasculature may be technically infeasible in such cases. Therefore, non-standard surgical approaches should be considered in liver transplant recipients.

In patients with end-stage liver disease, coagulopathy is often observed, and comorbidities may contribute to hypercoagulability. In rare cases, patients with systemic lupus erythematosus (SLE) may present with Budd-Chiari syndrome (BCS) as a manifestation of lupus-related antiphospholipid syndrome (APS), leading to large venous thrombosis. The presence of APS has been reported in 10–12% of cases of BCS [2]. Furthermore, the autoimmune hepatitis and primary biliary cholangitis (AIH-PBC) overlap syndrome has been reported with an incidence of approximately 2% in SLE patients with abnormal liver function tests [3]. To our knowledge, no studies have reported liver transplantation in patients with AIH-PBC overlap syndrome and lupus-related BCS to date.

Vascular thrombosis can be a challenging surgical factor requiring an individual approach. The selection of treatment options depends on the severity of the thrombosis, its characteristics, and the underlying disease. The initial management often consists of anticoagulation therapy, which effectively prevents thrombus propagation; however, it does not actively remove existing thrombosis [4]. Partial and local venous obstruction can be treated with thrombolysis therapy (systemic or catheter-directed) or endovascular mechanical thrombectomy [5]. If extensive venous thrombosis is observed, these methods may be insufficient, and surgical procedures might be nonexpendable.

Several surgical approaches have been described for managing incomplete IVC occlusion during LTx. One approach involves performing conventional LTx with resection of the thrombosed segment of the native IVC, followed by intraoperative thrombectomy if necessary [6]. Another strategy includes cavoplasty with a patch or a V–Y plasty technique [7,8]. In cases of complete IVC thrombosis, replacement of the affected part may be required. Previous studies have described the use of various conduits to perform this procedure, including the interposing autologous veins, cadaveric venous allografts, cadaveric aortic allografts, synthetic material, or a combination of synthetic material and autologous vein for IVC reconstruction [9,10,11,12]. However, some patients with extremely large IVC thrombosis may require LTx with graft-IVC anastomoses beyond the site of the obstruction. Therefore, an individual approach is necessary to select the most appropriate option.

## 2. Case Presentation

A 25-year-old woman with chronic liver failure related to AIH-PBC overlap syndrome with concomitant BCS was referred to the Department of General, Transplant, and Liver Surgery at the Medical University of Warsaw for the treatment of decompensated cirrhosis. The patient also had SLE that caused APS and chronic renal insufficiency. The patient was initially treated in the Rheumatology Department; however, due to deterioration of the gastrointestinal system, she was referred to the Gastroenterology Department, where management included hepatoprotective therapy with ursodeoxycholic acid, antibiotic prophylaxis, anticoagulation therapy, vasopressin analogues, encephalic prophylaxis and therapeutic paracentesis. A CT scan revealed an enlarged cirrhotic liver, with significant perfusion abnormalities typical of obstructed outflow. Ultrasound examination showed patent portal, middle, and left hepatic veins. The right hepatic vein was partially obstructed close to the caval confluence. The IVC was completely obstructed on account of thrombosis extending from both common iliac veins to the level of the right atrium. Posterior to the obstructed IVC, a heavily enlarged prevertebral venous network of the azygos-hemiazygos system was identified. Venous blood flow from the lower extremities and both kidneys was diverted to the superior vena cava through the azygos-hemiazygos veins and pre-vertebral collaterals.

**Figure 1:**
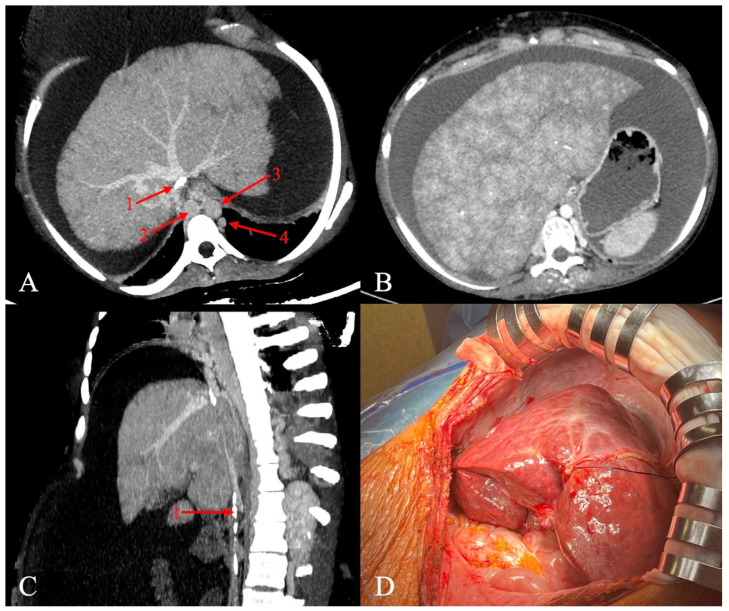
Pretransplant CT scans (**A**–**C**) and intraoperative picture of the recipient’s native liver (**D**). The pretransplant recipient CT scans show calcified IVC (red arrow 1; (**A**,**C**)) and dilated azygos (red arrow 2, (**A**)) and hemiazygos veins (red arrow 4, (**A**)), maintaining blood flow from the lower part of the body. The CT scan (nutmeg liver, (**B**)) and intraoperative picture (**D**) show perfusion abnormalities on account of chronic venous congestion. CT, computed tomography; IVC, inferior vena cava; red arrow 3, aorta.

The patient was accepted for deceased-donor liver transplantation after several attempts of thrombolytic therapy and radiological interventions. MELD-Na and Child-Pugh scores were 16 and 9 at the time of transplantation. Tense ascites and significantly developed collateral circulation were present. The veno-venous bypass was used. A total of 17 L of ascitic fluid was drained. Figure 1 shows hepatic congestion and venous abnormalities identified on prior CT scans.

**Figure 2:**
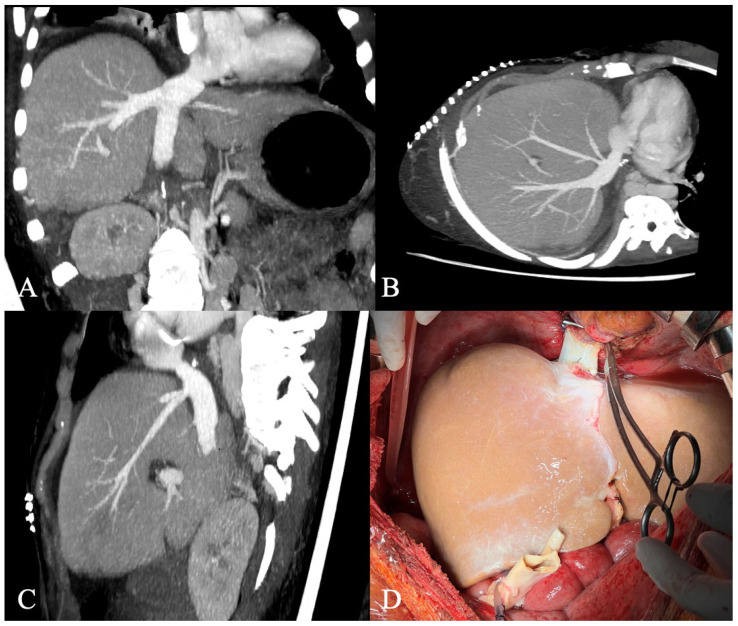
Posttransplant CT scans (**A**–**C**) and intraoperative picture of the IVC-atrial anastomosis (**D**). The pretransplant recipient CT scans show anastomosis of the donor IVC to the recipient’s right atrium (coronal plane, (**A**); axial plane (**B**), sagittal plane (**C**)). CT, computed tomography; IVC, inferior vena cava.

Extensive portosystemic collateral circulation was present, with a wide recanalized umbilical vein and diaphragmatic collaterals, which posed additional risk during the dissection of the intradiaphragmatic portion of the IVC. The azygos vein, which was the sole vessel maintaining blood flow to the heart, had to be preserved during this maneuver. After the removal of the recipient’s liver, it was confirmed that only the confluence of the IVC to the right atrium was patent. The pericardium was opened, and an anastomosis between the recipient’s right atrium and the graft’s suprahepatic segment of the IVC was performed using 4.0 polypropylene continuous suture (Supplementary Materials). The infrahepatic part of the donor’s IVC was left closed with the same suture technique. The outflow of the recipient’s infrahepatic IVC was maintained via the azygos and hemiazygos veins. Figure 2 shows the result of the IVC–atrial anastomosis. The portal, arterial and biliary anastomoses were performed using standard technique as described before [13]. The abdominal cavity was carefully checked before abdominal closure was attempted. No abnormalities of the gastrointestinal system were observed.

In the following days, a systematic improvement of the graft function was observed in laboratory tests (Table 1).

During the patient’s initial stay in the intensive care unit, the patient was conscious, respiratory stable, but hemodynamically unstable, requiring a low dosage of norepinephrine infusion for two days. Albumin supplementation was initiated, and broad-spectrum antibiotics and subcutaneous injection of low-molecular-weight heparin were administered during the post-transplant period. Immune suppression was achieved by use of the combination of tacrolimus, corticosteroid, and mycophenolate mofetil. The patient was maintained on oral feeding supplemented with parenteral nutrition. A series of follow-up post-transplant Doppler ultrasound examinations reported good venous patency with no disturbances in the hepatic and portal venous system. On the 10th day, the ultrasound Doppler revealed approximately 50 cm/s flow in the portal vein (PV) without flow distortion. The patient underwent a second laparotomy for a subdiaphragmatic hematoma and perforation of the cecum secondary to intestinal torsion. Intraoperative examination revealed 250 milliliters of hematoma behind the right liver lobe. The intestinal and cecum adhesions were observed in the abdominal cavity, as well as incomplete intestinal rotation to the right side. The intraoperative view seemed to indicate enteroparesis. The caecum was dilated up to 12 cm with a necrotic wall close to the local perforation. No abnormalities were observed in the hepatic or portal venous systems. The site of bleeding from the right diaphragmatic vein was patched and a right hemicolectomy was performed. Following this procedure, the patient remained intubated. Attempts at extubation were unsuccessful, requiring re-intubation. The follow-up imaging included abdominal ultrasound with Doppler flow assessment and computed tomography examinations. During this time, catecholamine infusion was maintained at low doses. On the 24th day, the patient underwent splenic artery embolization to reduce portal hypertension and the development of ascites with satisfactory effect. On the 32nd day, tracheostomy was performed due to prolonged mechanical ventilation. The patient was alert, receiving assisted ventilation through tracheostomy. Hemodynamic instability persisted, requiring low-dose norepinephrine. Two weeks later, the patient underwent a third laparotomy for recurrent intestinal adhesions with intestinal obstruction. Intraoperative examination revealed massive adhesion close to the previous intestinal anastomosis, which was the cause of the obstruction. No anomalies were observed in the portal system or hepatic vasculature. Despite normalization of liver function tests, the patient’s condition deteriorated, and the patient died 56 days after LTx.

## 3. Discussion

Complete IVC thrombosis represents one of the most challenging aspects of LTx for patients with BCS. Such rare situations necessitate non-standard solutions; however, the site of the hepatic venous outflow reconstruction does not necessarily have to be in the IVC. In our patient, anatomical anomalies enabled the operation due to enlarged azygos-hemiazygos veins and pre-vertebral collaterals, which drained blood from the lower part of the body. As discussed by Gonultas et al., maintaining venous continuity is essential for patients who lack a well-developed venous collateral circulation system or exhibit insufficient venous drainage [14]. Furthermore, Karaca et al. suggested that graft anastomosis should not be performed to an occluded IVC when drainage of the lower extremities and kidneys is already established through the aforementioned collateral systems [15]. Patients with well-developed venous collaterals may be suitable candidates for IVC anastomosis limited to outflow reconstruction at the level of the suprahepatic IVC, thereby obviating the need for complete anatomical restoration. In such cases, it is possible to anastomose the IVC of the liver graft to any patent segment of the recipient’s IVC above the critical stenosis. Alternatively, if the veno-venous anastomosis is not feasible, an anastomosis between the suprahepatic IVC of the graft to the recipient’s right atrium may be performed. In both scenarios, it is frequently necessary to achieve adequate access by performing a sternotomy or transdiaphragmatic approach. Yoon YI et al. emphasize the need for caution during partial clamping of the pulsating right atrium, as the thin and shallow atrial tissue is highly susceptible to clamp slippage during surgery. A recommended strategy involves obtaining sufficient space and visualization through a lower hemisternotomy, combined with the use of Allis tissue forceps to secure both corners of the clamped atrium under a Satinsky clamp, thereby minimizing the risk of slippage [16]. We propose three advantageous modifications in liver transplantation involving IVC-atrial anastomosis. Access to the right atrium should be achieved via a transdiaphragmatic approach, avoiding a thoracotomy. In our case, a wide recanalized umbilical vein and diaphragmatic collaterals posed additional risk during the dissection of the intradiaphragmatic portion of the IVC. Therefore, particular caution is required during this maneuver. To facilitate anastomosis and accurate anatomical positioning of the graft, sufficient length of the suprahepatic IVC must be preserved. To avoid dissection near the thrombosed IVC in the recipient, the graft infrahepatic IVC may be left closed without anatomical anastomosis, provided that collateral outflow from the recipient’s infrahepatic IVC is maintained. Table 2 summarizes the studies with a direct venous outflow reconstruction to the right atrium. Adequate collateral circulation may potentially eliminate the need for utilization of the veno-venous bypass; however, our patient was hemodynamically unstable, making the use of extracorporeal veno-venous bypass a safer option.

For patients with end-stage liver disease, liver transplantation remains the only curative option with the potential for full recovery. However, autoimmune diseases, such as APS and PBC-AIH overlap syndrome, may contribute to a higher risk of failure. The mortality rate of patients with APS is 50–80% higher than that of the general population [23]. Furthermore, compared to PBC patients alone, patients with PBC-AIH overlap syndrome have shown higher rates of mortality associated with more cirrhosis-related complications, such as symptomatic portal hypertension, esophageal varices, gastrointestinal bleeding, and ascites [24]. Additional prevention focused on antithrombotic events should be implemented. It is recommended to use a quick reintroduction of anticoagulation, although this must be balanced against the increased risk of bleeding after an operation [25]. In our study, the subcutaneous low-molecular-weight heparin was administered to the patient during the post-transplant period. In the literature, authors reported different anticoagulation therapies after LTx in patients with APS, including heparin, rivaroxaban, or dabigatran etexilate [26,27,28,29]. Further studies are needed to determine the optimal anticoagulant therapy in patients with APS.

In our case, the patient survived the early postoperative period (<30 days) with satisfactory improvement of graft function despite other complications. We did not observe any disturbances in the hepatic and portal venous system during the post-transplant period, other than portal hypertension. Unfortunately, the patient died at the end of the second month after transplantation. It is difficult to unambiguously certify whether the death could be related to the liver transplantation itself or a consequence of the complexity of pre-existing conditions, multiple comorbidities, and several post-operative complications. However, none of these complications were directly related to the function of the graft and surgical technique.

## 4. Conclusions

Vascular abnormalities with extensive thrombosis pose a significant problem in patients with end-stage liver disease and may limit their access to liver transplantation. The surgical approach in these patients requires a comprehensive analysis of the vascular anatomy with emphasis on the restoration of functional hemodynamics after the transplantation. Non-standard liver transplantation techniques, such as an IVC-atrial anastomosis and partial reconstruction of IVC anatomy, may be required and implemented in patients with severely developed collateral circulation. This kind of anatomical abnormality, despite its complexity, should not be considered an absolute contraindication to liver transplantation.

## Figures and Tables

**Table 1:** Postoperative laboratory tests results.

Time After Surgery (Days)								
Laboratory Test	0	1	2	3	5	10	15	30
ALT (U/L)	246	417	387	176	143	69	14	8
AST (U/L)	1431	2196	1507	509	56	148	26	28
Albumin (g/dL)	2.7	2.7	1.9	1.8	2	2.9	3.1	4
Bilirubin (mg/dL)	1.55	2.39	2.64	2.51	3.53	1.55	1.03	2.16
LDH (U/L)	1281	873	479	212	133	148	128	195
CRP (mg/dL)	46.7	29.3	39.7	17.4	9.3	200	95.1	174
Hemoglobin (g/dL)	8.2	12	11	8.7	10	10.6	8.3	9.5
PT-INR	2.07	2.23	2.74	2.78	1.67	1.46	1.05	1.08
Creatinine (mg/dL)	1.21	1.55	1.53	1.59	1.18	1.29	1.48	1.31

ALT, alanine aminotransferase; AST, aspartate aminotransferase; LDH, lactate dehydrogenase; CRP, C-reactive protein; PT-INR, prothrombin time-international normalized ratio.

**Table 2:** Summary of reported cases of hepato-atrial anastomosis in adult liver transplant recipients.

Type of Liver Transplantation (Number)	Etiology (Number)	Veno-Venous Bypass Use	Congenital Absence of IVC	Range of Obstruction	Pre-Surgical Treatment	Venous Reconstruction Technique	References
Living donor (1)	BCS, HCC	No	No	Hepatic veins and retrohepatic IVC	Mesoatrial shunt with synthetic graft	Synthetic IVC graft	Choi GS et al. Transplant Proc. 2010 [17]
Living donor (1)	BCS	No	No	Hepatic veins and patrial suprahepatic IVC	Not described	Cryopreserved IVC graft	Yalak F et al. Int J Organ Transplant Med. 2015 [18]
Living donor (2)	HCC, PSC	No	Yes	Not described	Not described	The RHV, MHV and LHV were joined in a single orifice and anastomosed to the suprahepatic IVC of the new liver	Angelico R et al. Int J Surg. 2015 [19]
Deceased donor (1)	BCS	Yes	No	RHV and MHV, and complete supra- and infrahepatic IVC.	Not described	Anastomosis between graft’s suprahepatic IVC and recipient’s thoracic IVC. The graft’s infrahepatic IVC was suture-closed as a blind pouch	Hong SY et al. Transplant Proc. 2017 [20]
Deceased donor (1)	BCS, Hepatic Alveolar Echinococcosis	Yes	No	MHV and LHV	Albendazole therapy	Direct anastomosis of the graft’s suprahepatic IVC and the right atrium. The graft’s infrahepatic IVC was anastomosed to the recipient’s IVC	Kobryń K et al. BMC Surg. 2017 [21]
Living donor (1)	BCS	No	No	Hepatic veins and retrohepatic IVC	Percutaneous thrombectomy and stent placement	Direct anastomosis of the graft’s RHV and the right atrium	Sabra TA et al. Int J Surg Case Rep. 2018 [22]
Living donor (3)	BCS (3)	No	No	Hepatic veins, and complete supra- and infrahepatic IVC	Anticoagulants	Direct anastomosis of the graft’s RHV and the right atrium; iliac vein conduit from a deceased donor was joined to the new liver and anastomosed to the right atrium	Karaca CA et al. Exp Clin Transplant. 2019 [15]
Living donor (5)	BCS (5), HCC (3), MOVC (2)	No	No	Hepatic veins, and retro- and supra hepatic IVC	Not described	Synthetic IVC graft	Yoon YI et al. Ann Surg. 2019 [16]
Deceased donor (1)	AIH-PBC, BCS, SLE	Yes	No	Hepatic veins, and complete supra- and infrahepatic IVC	Antithrombotic therapy and balloon angioplasty	Direct anastomosis of the graft’s suprahepatic IVC and the right atrium. The graft’s infrahepatic IVC suture-closed as a blind pouch	Present study

IVC, inferior vena cava; BCS, Budd-Chiari syndrome; HCC, hepatocellular carcinoma; PSC, primary sclerosis cholangitis; RHV, right hepatic vein; LHV, left hepatic vein; MHV, middle hepatic vein; MOVC, membranous obstruction of the inferior vena cava; AIH-PBC, autoimmune hepatitis and primary biliary cholangitis; SLE, systemic lupus erythematosus.

## Data Availability

Data are contained within this article.

## Supplementary Materials

The following supporting information can be downloaded at: https://www.mdpi.com/article/10.3390/jcm15010384/s1, Video S1: IVC-atrial anastomosis.

## Abbreviations

The following abbreviations are used in this manuscript:

AIH-PBC	autoimmune hepatitis and primary biliary cholangitis
ALT	alanine aminotransferase
APS	antiphospholipid syndrome
AST	aspartate aminotransferase
BCS	Budd-Chiari syndrome
CRP	c-reactive protein
HCC	hepatocellular carcinoma
IVC	inferior vena cava
LDH	lactate dehydrogenase
LHV	left hepatic vein
LTx	liver transplantation
MELD-Na	model for end-stage liver disease
MHV	middle hepatic vein
MOVC	membranous obstruction of the inferior vena cava
PSC	primary sclerosing cholangitis
PT-INR	prothrombin time-international normalized ratio
RHV	right hepatic vein
SLE	systemic lupus erythematosus
